# Diaqua­bis­[bis­(pyrazin-2-yl) sulfide-κ*N* ^4^]bis­(thio­cyanato-κ*N*)iron(II) monohydrate

**DOI:** 10.1107/S1600536813006314

**Published:** 2013-03-09

**Authors:** Susanne Wöhlert, Inke Jess, Christian Näther

**Affiliations:** a Institut für Anorganische Chemie, Christian-Albrechts-Universität Kiel, Max-Eyth-Strasse 2, 24118 Kiel, Germany

## Abstract

In the title compound [Fe(NCS)_2_(C_8_H_6_N_4_S)_2_(H_2_O)_2_]·H_2_O, the Fe^II^ cation is coordinated by two *N*-bonded thio­cyanate anions, two *N* ^4^-bonded bis­(pyrazin-2-yl) sulfide ligands and two water mol­ecules in an slightly distorted octa­hedral geometry. The Fe^II^ cation is located on a center of inversion and the lattice water mol­ecule on a twofold rotation axis. The thio­cyanate anions, the coordinating water mol­ecules and the sulfide ligands occupy general positions. The complex mol­ecules and lattice water mol­ecules are linked into a three-dimensional network by O—H—N and O—H⋯O hydrogen bonds.

## Related literature

For the background to this work, see: Wöhlert & Näther (2013 ▶).
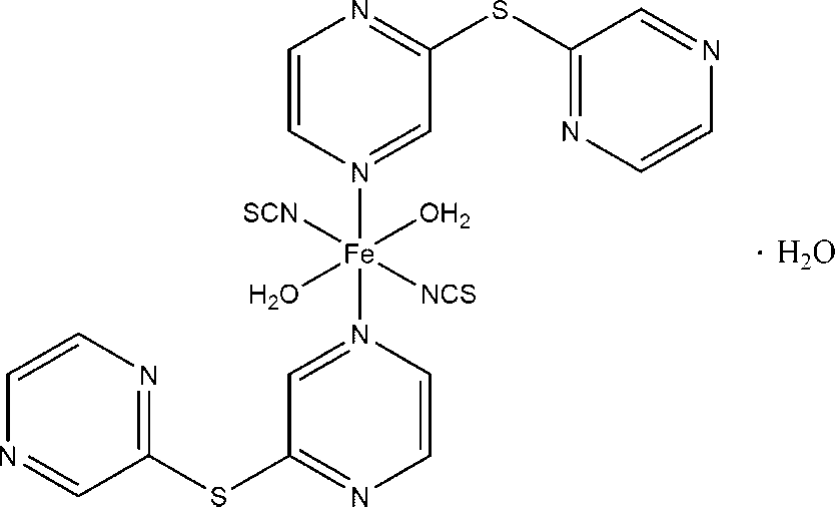


## Experimental

### 

#### Crystal data

[Fe(NCS)_2_(C_8_H_6_N_4_S)_2_(H_2_O)_2_]·H_2_O
*M* *_r_* = 606.51
Monoclinic, 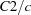

*a* = 11.5110 (8) Å
*b* = 15.8583 (9) Å
*c* = 14.8025 (12) Å
β = 109.770 (8)°
*V* = 2542.9 (3) Å^3^
*Z* = 4
Mo *K*α radiation
μ = 0.96 mm^−1^
*T* = 200 K
0.25 × 0.15 × 0.09 mm

#### Data collection

Stoe IPDS-1 diffractometer
Absorption correction: numerical (*X-SHAPE* and *X-RED32*; Stoe & Cie, 2008 ▶) *T*_min_ = 0.690, *T*_max_ = 0.859
10774 measured reflections
2496 independent reflections
2012 reflections with *I* > 2σ(*I*)
*R*_int_ = 0.044

#### Refinement

*R*[*F* ^2^ > 2σ(*F* ^2^)] = 0.038
*wR*(*F* ^2^) = 0.090
*S* = 1.05
2496 reflections
165 parameters
H-atom parameters constrained
Δρ_max_ = 0.52 e Å^−3^
Δρ_min_ = −0.49 e Å^−3^

### 

Data collection: *X-AREA* (Stoe & Cie, 2008 ▶); cell refinement: *X-AREA*; data reduction: *X-AREA*; program(s) used to solve structure: *SHELXS97* (Sheldrick, 2008 ▶); program(s) used to refine structure: *SHELXL97* (Sheldrick, 2008 ▶); molecular graphics: *XP* in *SHELXTL* (Sheldrick, 2008 ▶) and *DIAMOND* (Brandenburg, 2011 ▶); software used to prepare material for publication: *XCIF* in *SHELXTL* and *publCIF* (Westrip, 2010 ▶).

## Supplementary Material

Crystal structure: contains datablock(s) I, global. DOI: 10.1107/S1600536813006314/bt6895sup1.cif

Structure factors: contains datablock(s) I. DOI: 10.1107/S1600536813006314/bt6895Isup2.hkl

Additional supplementary materials:  crystallographic information; 3D view; checkCIF report

## Figures and Tables

**Table 1:** Selected bond lengths (Å)

Fe1—O1	2.0965 (17)
Fe1—N1	2.101 (2)
Fe1—N10	2.235 (2)

**Table 2:** Hydrogen-bond geometry (Å, °)

*D*—H⋯*A*	*D*—H	H⋯*A*	*D*⋯*A*	*D*—H⋯*A*
O2—H1*O*2⋯N11	0.84	2.00	2.834 (3)	171
O1—H1*O*1⋯N20^i^	0.84	1.94	2.765 (3)	165
O1—H2*O*1⋯O2^ii^	0.84	1.94	2.749 (3)	162

Symmetry codes: (i) 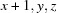; (ii) 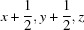.

**Fig. 1.**
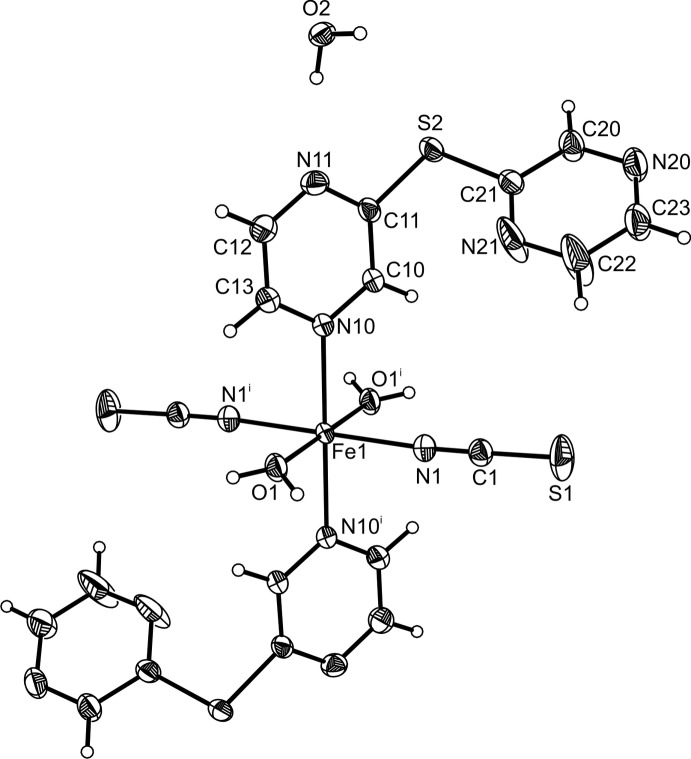
Crystal structure of the title compound with labeling and displacement ellipsoids drawn at the 50% probability level. Symmetry code: i = -x + 3/2, -y + 3/2, -z.

**Fig. 2.**
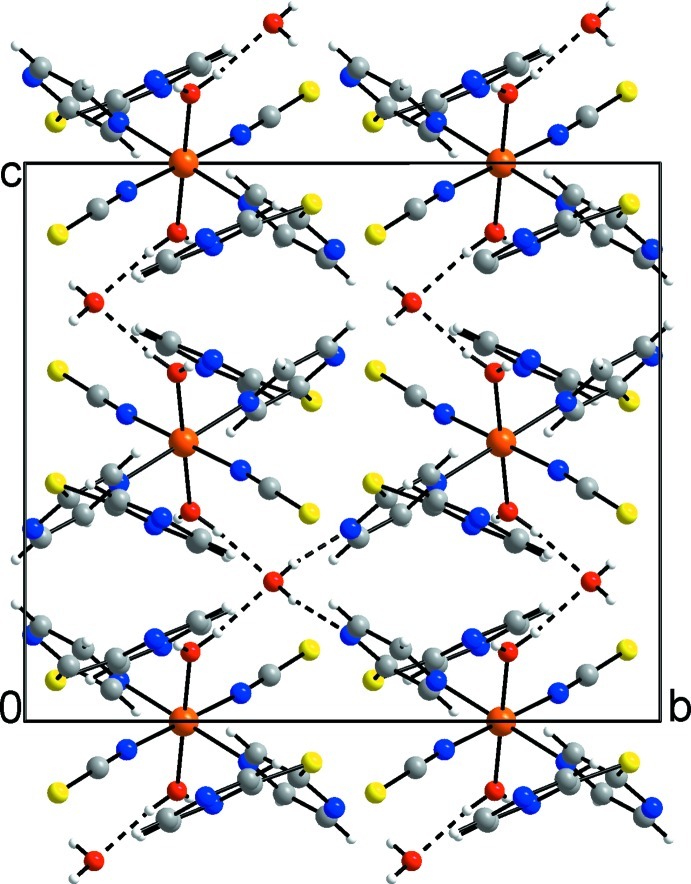
Crystal structure of the title compound with view along the a-axis (orange = iron, blue = nitrogen, yellow = sulfur, red = oxygen, grey = carbon, white = hydrogen). Intermolecular hydrogen bonding is shown as dashed lines.

## supplementary crystallographic information

### Comment

Recently we have reported on the synthesis and characterization of transition metal thiocyanato compounds with 2-chloropyrazine as neutral co-ligand (Wöhlert & Näther, 2013). In the course of these investigations, we have reacted iron(II)sulfate with potassium thiocyanate and 2-chloropyrazine under hydrothermal conditions, which accidently lead to the formation of single crystals of the title compound that were characterized by single-crystal X-ray diffraction. In the crystal structure, each iron(II) cation is coordinated by two *N*-bonded thiocyanato anions, two 2,2'-dipyrazinesulfide ligands and two water molecules within a slightly distorted octahedra (Fig.1 and Tab. 1). The Fe—N and Fe—O distances range from 2.096 5(17) Å to 2.235 (2) Å with angles arround the iron(II) cation between 87.98 (8) ° to 92.02 (8) ° and of 180 ° (Tab. 1). The asymmetric unit consists of one iron(II) cation located on a center of inversion, one water molecule on a 2-fold axis as well as of one 2,2'-dipyrazinesulfide ligand, one thiocyanato anion and one water molecule all of them located in general position. The discrete complexes are connected by the non-coordinating water molecules into a three-dimensional network through intermolecular O—H—N and O—H—O hydrogen bonding (Fig. 2). In this arrangement each non-coordinating water molecule acts as acceptor in two O—H—O hydrogen bonds and as a donor in two O—H—N hydrogen bonds (Fig. 2 and Tab.2).

### Experimental

FeSO_4_x7H_2_O, KNCS and 2-chloropyrazine were obtained from Alfa Aesar. All chemicals were used without further purification. 0.15 mmol (41.7 mg) FeSO_4_x7H_2_O, 0.3 mmol (29.1 mg) KNCS and 0.3 mmol (26.4 µ*L*) 2-chloropyrazine were reacted in 1 ml water in a closed test-tube at 120 °C for 3 days. Red single crystals of the title compound were obtained after two days on cooling.

### Refinement

All C—H H atoms were located in difference map but were positioned with idealized geometry and were refined isotropic with *U*_iso_(H) = 1.2 *U*_eq_(C) using a riding model with C—H = 0.95 Å for aromatic H atoms. The water H atoms were located in a difference map, their bond lenghts were set to ideal values of 0.84 Å and finally they were refined using a riding model with *U*_iso_(H) = 1.5 *U*_eq_(O).

### Crystal data

**Table:**

[Fe(NCS)_2_(C_8_H_6_N_4_S)_2_(H_2_O)_2_]·H_2_O	*F*(000) = 1240
*M**_r_* = 606.51	*D*_x_ = 1.584 Mg m^−^^3^
Monoclinic, *C*2/*c*	Mo *K*α radiation, λ = 0.71073 Å
Hall symbol: -C 2yc	Cell parameters from 10774 reflections
*a* = 11.5110 (8) Å	θ = 2.3–26.0°
*b* = 15.8583 (9) Å	µ = 0.96 mm^−^^1^
*c* = 14.8025 (12) Å	*T* = 200 K
β = 109.770 (8)°	Block, red
*V* = 2542.9 (3) Å^3^	0.25 × 0.15 × 0.09 mm
*Z* = 4	

### Data collection

**Table:**

Stoe IPDS-1 diffractometer	2496 independent reflections
Radiation source: fine-focus sealed tube	2012 reflections with *I* > 2σ(*I*)
Graphite monochromator	*R*_int_ = 0.044
phi scan	θ_max_ = 26.0°, θ_min_ = 2.3°
Absorption correction: numerical (*X-SHAPE* and *X-RED32*; Stoe & Cie, 2008)	*h* = −14→13
*T*_min_ = 0.690, *T*_max_ = 0.859	*k* = −19→19
10774 measured reflections	*l* = −18→18

### Refinement

**Table:**

Refinement on *F*^2^	Primary atom site location: structure-invariant direct methods
Least-squares matrix: full	Secondary atom site location: difference Fourier map
*R*[*F*^2^ > 2σ(*F*^2^)] = 0.038	Hydrogen site location: inferred from neighbouring sites
*wR*(*F*^2^) = 0.090	H-atom parameters constrained
*S* = 1.05	*w* = 1/[σ^2^(*F*_o_^2^) + (0.0412*P*)^2^ + 5.1033*P*] where *P* = (*F*_o_^2^ + 2*F*_c_^2^)/3
2496 reflections	(Δ/σ)_max_ < 0.001
165 parameters	Δρ_max_ = 0.52 e Å^−^^3^
0 restraints	Δρ_min_ = −0.49 e Å^−^^3^

### Special details

**Table:**

Geometry. All e.s.d.'s (except the e.s.d. in the dihedral angle between two l.s. planes) are estimated using the full covariance matrix. The cell e.s.d.'s are taken into account individually in the estimation of e.s.d.'s in distances, angles and torsion angles; correlations between e.s.d.'s in cell parameters are only used when they are defined by crystal symmetry. An approximate (isotropic) treatment of cell e.s.d.'s is used for estimating e.s.d.'s involving l.s. planes.
Refinement. Refinement of *F*^2^ against ALL reflections. The weighted *R*-factor *wR* and goodness of fit *S* are based on *F*^2^, conventional *R*-factors *R* are based on *F*, with *F* set to zero for negative *F*^2^. The threshold expression of *F*^2^ > σ(*F*^2^) is used only for calculating *R*-factors(gt) *etc*. and is not relevant to the choice of reflections for refinement. *R*-factors based on *F*^2^ are statistically about twice as large as those based on *F*, and *R*- factors based on ALL data will be even larger.

### Fractional atomic coordinates and isotropic or equivalent isotropic displacement parameters (Å^2^)

**Table:**

	*x*	*y*	*z*	*U*_iso_*/*U*_eq_	
Fe1	0.7500	0.7500	0.0000	0.01850 (14)	
N1	0.6524 (2)	0.83973 (14)	0.05049 (17)	0.0283 (5)	
C1	0.5978 (2)	0.88512 (16)	0.08281 (19)	0.0250 (6)	
S1	0.51942 (8)	0.94785 (6)	0.12789 (7)	0.0495 (3)	
N10	0.68155 (19)	0.64769 (13)	0.07296 (16)	0.0217 (4)	
C10	0.5612 (2)	0.63655 (16)	0.05785 (19)	0.0231 (5)	
H10	0.5030	0.6753	0.0184	0.028*	
C11	0.5199 (2)	0.56900 (16)	0.09904 (19)	0.0221 (5)	
C12	0.7178 (3)	0.52477 (18)	0.1702 (2)	0.0309 (6)	
H12	0.7760	0.4863	0.2100	0.037*	
C13	0.7596 (2)	0.59152 (17)	0.1297 (2)	0.0275 (6)	
H13	0.8458	0.5978	0.1424	0.033*	
N11	0.5975 (2)	0.51276 (14)	0.15487 (16)	0.0265 (5)	
S2	0.36039 (6)	0.54851 (4)	0.07358 (6)	0.03024 (18)	
N20	0.1422 (2)	0.71530 (17)	0.13551 (19)	0.0365 (6)	
C20	0.1863 (2)	0.64829 (19)	0.1039 (2)	0.0323 (6)	
H20	0.1322	0.6031	0.0752	0.039*	
C21	0.3091 (2)	0.64298 (17)	0.1120 (2)	0.0264 (6)	
C22	0.3399 (4)	0.7716 (3)	0.1788 (6)	0.129 (3)	
H22	0.3930	0.8179	0.2049	0.155*	
C23	0.2211 (3)	0.7764 (2)	0.1745 (3)	0.0604 (12)	
H23	0.1938	0.8244	0.2000	0.072*	
N21	0.3859 (3)	0.7044 (2)	0.1476 (4)	0.0998 (19)	
O1	0.90576 (16)	0.76058 (11)	0.12427 (13)	0.0264 (4)	
H1O1	0.9775	0.7416	0.1362	0.040*	
H2O1	0.9184	0.8036	0.1593	0.040*	
O2	0.5000	0.39109 (15)	0.2500	0.0247 (5)	
H1O2	0.5302	0.4227	0.2182	0.037*	

### Atomic displacement parameters (Å^2^)

**Table:**

	*U*^11^	*U*^22^	*U*^33^	*U*^12^	*U*^13^	*U*^23^
Fe1	0.0136 (2)	0.0178 (2)	0.0259 (3)	0.00060 (19)	0.00904 (18)	−0.0007 (2)
N1	0.0268 (12)	0.0247 (11)	0.0384 (13)	0.0029 (9)	0.0175 (11)	−0.0019 (10)
C1	0.0210 (13)	0.0266 (13)	0.0261 (13)	0.0013 (11)	0.0064 (11)	−0.0026 (11)
S1	0.0410 (5)	0.0572 (5)	0.0516 (5)	0.0152 (4)	0.0173 (4)	−0.0235 (4)
N10	0.0191 (10)	0.0198 (10)	0.0292 (11)	0.0009 (8)	0.0118 (9)	0.0004 (9)
C10	0.0185 (12)	0.0223 (12)	0.0305 (14)	0.0012 (10)	0.0110 (10)	0.0006 (10)
C11	0.0210 (12)	0.0224 (12)	0.0266 (13)	−0.0022 (10)	0.0129 (10)	−0.0066 (10)
C12	0.0261 (14)	0.0291 (14)	0.0359 (15)	0.0035 (11)	0.0083 (12)	0.0092 (12)
C13	0.0193 (12)	0.0259 (13)	0.0369 (15)	0.0013 (10)	0.0091 (11)	0.0041 (11)
N11	0.0298 (12)	0.0236 (11)	0.0283 (12)	−0.0014 (9)	0.0128 (10)	0.0030 (9)
S2	0.0229 (3)	0.0244 (3)	0.0485 (4)	−0.0060 (3)	0.0187 (3)	−0.0088 (3)
N20	0.0220 (12)	0.0396 (14)	0.0479 (15)	0.0046 (11)	0.0118 (11)	−0.0067 (12)
C20	0.0200 (13)	0.0348 (15)	0.0402 (16)	−0.0011 (11)	0.0075 (12)	−0.0097 (12)
C21	0.0240 (13)	0.0259 (13)	0.0332 (14)	−0.0038 (11)	0.0149 (11)	−0.0040 (11)
C22	0.058 (3)	0.081 (3)	0.285 (9)	−0.046 (3)	0.104 (4)	−0.123 (5)
C23	0.043 (2)	0.0392 (18)	0.115 (4)	−0.0091 (15)	0.046 (2)	−0.034 (2)
N21	0.0447 (19)	0.068 (2)	0.215 (5)	−0.0369 (17)	0.080 (3)	−0.092 (3)
O1	0.0163 (8)	0.0269 (9)	0.0335 (10)	0.0015 (7)	0.0054 (7)	−0.0062 (8)
O2	0.0325 (14)	0.0181 (12)	0.0282 (14)	0.000	0.0166 (11)	0.000

### Geometric parameters (Å, º)

**Table:**

Fe1—O1^i^	2.0965 (17)	C12—H12	0.9500
Fe1—O1	2.0965 (17)	C13—H13	0.9500
Fe1—N1	2.101 (2)	S2—C21	1.773 (3)
Fe1—N1^i^	2.101 (2)	N20—C23	1.319 (4)
Fe1—N10	2.235 (2)	N20—C20	1.329 (4)
Fe1—N10^i^	2.235 (2)	C20—C21	1.381 (4)
N1—C1	1.160 (3)	C20—H20	0.9500
C1—S1	1.630 (3)	C21—N21	1.302 (4)
N10—C10	1.339 (3)	C22—N21	1.340 (5)
N10—C13	1.340 (3)	C22—C23	1.349 (5)
C10—C11	1.393 (4)	C22—H22	0.9500
C10—H10	0.9500	C23—H23	0.9500
C11—N11	1.333 (3)	O1—H1O1	0.8400
C11—S2	1.775 (3)	O1—H2O1	0.8400
C12—N11	1.339 (4)	O2—H1O2	0.8400
C12—C13	1.381 (4)		
			
O1^i^—Fe1—O1	180.0	N11—C12—C13	121.9 (2)
O1^i^—Fe1—N1	87.98 (8)	N11—C12—H12	119.1
O1—Fe1—N1	92.02 (8)	C13—C12—H12	119.1
O1^i^—Fe1—N1^i^	92.02 (8)	N10—C13—C12	121.5 (2)
O1—Fe1—N1^i^	87.98 (8)	N10—C13—H13	119.2
N1—Fe1—N1^i^	180.00 (12)	C12—C13—H13	119.2
O1^i^—Fe1—N10	91.68 (8)	C11—N11—C12	116.6 (2)
O1—Fe1—N10	88.32 (8)	C21—S2—C11	102.11 (12)
N1—Fe1—N10	90.08 (8)	C23—N20—C20	116.9 (3)
N1^i^—Fe1—N10	89.92 (8)	N20—C20—C21	121.3 (3)
O1^i^—Fe1—N10^i^	88.32 (8)	N20—C20—H20	119.3
O1—Fe1—N10^i^	91.68 (8)	C21—C20—H20	119.3
N1—Fe1—N10^i^	89.92 (8)	N21—C21—C20	121.6 (3)
N1^i^—Fe1—N10^i^	90.08 (8)	N21—C21—S2	120.6 (2)
N10—Fe1—N10^i^	180.00 (9)	C20—C21—S2	117.8 (2)
C1—N1—Fe1	175.3 (2)	N21—C22—C23	122.7 (3)
N1—C1—S1	179.1 (2)	N21—C22—H22	118.6
C10—N10—C13	117.0 (2)	C23—C22—H22	118.6
C10—N10—Fe1	121.96 (17)	N20—C23—C22	121.1 (3)
C13—N10—Fe1	120.93 (17)	N20—C23—H23	119.5
N10—C10—C11	121.0 (2)	C22—C23—H23	119.5
N10—C10—H10	119.5	C21—N21—C22	116.2 (3)
C11—C10—H10	119.5	Fe1—O1—H1O1	130.1
N11—C11—C10	122.0 (2)	Fe1—O1—H2O1	121.7
N11—C11—S2	115.92 (19)	H1O1—O1—H2O1	102.0
C10—C11—S2	121.9 (2)		

Symmetry code: (i) −*x*+3/2, −*y*+3/2, −*z*.

### Hydrogen-bond geometry (Å, º)

**Table:**

*D*—H···*A*	*D*—H	H···*A*	*D*···*A*	*D*—H···*A*
O2—H1*O*2···N11	0.84	2.00	2.834 (3)	171
O1—H1*O*1···N20^ii^	0.84	1.94	2.765 (3)	165
O1—H2*O*1···O2^iii^	0.84	1.94	2.749 (3)	162

Symmetry codes: (ii) *x*+1, *y*, *z*; (iii) *x*+1/2, *y*+1/2, *z*.
